# Innate Immunity and Pathogenesis of Biliary Atresia

**DOI:** 10.3389/fimmu.2020.00329

**Published:** 2020-02-25

**Authors:** Ana Ortiz-Perez, Bryan Donnelly, Haley Temple, Greg Tiao, Ruchi Bansal, Sujit Kumar Mohanty

**Affiliations:** ^1^Department of Biomaterials Science and Technology, Technical Medical Centre, Faculty of Science and Technology, University of Twente, Enschede, Netherlands; ^2^Department of Pediatric and Thoracic Surgery, Cincinnati Children's Hospital Medical Center, Cincinnati, OH, United States

**Keywords:** biliary atresia, liver fibrosis, rotavirus, innate immunity, macrophages

## Abstract

Biliary atresia (BA) is a devastating fibro-inflammatory disease characterized by the obstruction of extrahepatic and intrahepatic bile ducts in infants that can have fatal consequences, when not treated in a timely manner. It is the most common indication of pediatric liver transplantation worldwide and the development of new therapies, to alleviate the need of surgical intervention, has been hindered due to its complexity and lack of understanding of the disease pathogenesis. For that reason, significant efforts have been made toward the development of experimental models and strategies to understand the etiology and disease mechanisms and to identify novel therapeutic targets. The only characterized model of BA, using a Rhesus Rotavirus Type A infection of newborn BALB/c mice, has enabled the identification of key cellular and molecular targets involved in epithelial injury and duct obstruction. However, the establishment of an unleashed chronic inflammation followed by a progressive pathological wound healing process remains poorly understood. Like T cells, macrophages can adopt different functional programs [pro-inflammatory (M1) and resolutive (M2) macrophages] and influence the surrounding cytokine environment and the cell response to injury. In this review, we provide an overview of the immunopathogenesis of BA, discuss the implication of innate immunity in the disease pathogenesis and highlight their suitability as therapeutic targets.

## Introduction

Biliary atresia (BA) is a devastating obliterative cholangiopathy that affects exclusively infants and is characterized by a progressive fibro-inflammatory obstruction of the extrahepatic and intrahepatic bile ducts that can lead to cirrhosis and liver failure (1–4). BA occurs in 1 out of 15,000 births in the US (5), affecting all ethnic groups, (6) and with a higher frequency in girls (7). Despite its low incidence, BA is the most common cause of neonatal cholestasis (3), end-stage liver disease in children and the number one indication of pediatric liver transplant worldwide (8, 9). The first disease symptoms include jaundice, alcoholic stools, dark urines (3), and high levels of serum bilirubin (10). A *conclusive* diagnosis of BA is based on an exploratory surgery where obstruction of the extrahepatic biliary tree can be observed and confirmed by a histological analysis of liver or biliary tissue biopsy (3). At the time of diagnosis, about 60 days of life on average (4), the obstructed extrahepatic remnants are removed and hepatoportoenterostomy (HPE, called Kasai) is performed to restore the bile flow (11). However, even if the Kasai procedure is performed during the first month of life and the cholestasis is resolved, bile duct proliferation, and fibrosis persist (9) resulting in the development of variable degrees of liver fibrosis, cirrhosis, portal hypertension, or other severe hepatic complications (12). Notably, the long-term survival of BA patients has extraordinarily improved in the last decades—from 70% in the 1990s to 80–90% in 2009 (13)—but the treatment still relies on surgery (HPE, transplantation), which is palliative, thereby highlighting the necessity of developing novel targeted therapies to prevent or reverse liver injury.

## Classification and Molecular Signatures

Traditionally, BA patients were divided into “embryonic/developmental” BA (<20%) and “perinatal/acquired” BA (> 80%) depending on their onset (14–16). The former is believed to originate during the first trimester of pregnancy and the accompanying clinical features suggest a developmental origin (4), the latter is thought to appear shortly after birth when the first symptoms become recognizable (10). The presence of splenic malformations—polysplenia but also asplenia—is characteristic of the Biliary Atresia Splenic Malformation (BASM) syndrome, the most representative form of embryonic BA (about 10%). The infants within this group were found to have a worse prognosis than infants with isolated BA (17). The remaining sub-group comprises patients with at least one non-splenic malformation. This group is also often included in the category of non-syndromic BA, since the presence of the underlying defects does not necessarily worsen the disease or implicates different mechanisms of pathogenesis (11, 18). Notably, BASM patients may also have another concomitant defect, such as cardiovascular and laterality defects (17).

In 2012, Davenport proposed the latest reference classification incorporating the cytomegalovirus (CMV)-associated and cystic BA variants to the aforementioned non-syndromic BA and BASM groups (19). CMV-associated BA refers to a subgroup of infants whose liver biopsies stained positive for immunoglobulin M (IgM) antibodies against CMV. The presence of these antibodies has been linked to the poorest HPE outcome and highest mortality, and the tissue biopsies revealed an exacerbated pro-inflammatory response (20): the predominant cellular profile observed in most of the BA patients (16). By contrast, cystic BA, an anatomic variant in which a cyst is formed close to the site of obstruction and a Th2-response is primed, was associated with an improved drainage after HPE and a better long-term outcome (21).

## Etiology

The etiology of BA is heterogeneous and has not been fully elucidated yet. Diverse theories regarding the causes of the disease have been formulated, including embryonic or developmental abnormalities (17, 21), exposure to exogenous triggers such as viruses or toxins (16, 22), immune immaturity (11, 23), immune dysregulation (24, 25), and autoimmunity (26–29). Furthermore, numerous susceptibility factors—such as genetic predisposition (30), maternal diabetes (17), or microchimerism (31)—have also been implicated in the pathogenesis of the disease. This complex cocktail of variables and factors supports the claim that biliary atresia is not a disease with a single etiology but a combination of different phenotypes that share certain clinical features, such as the obliteration of the biliary tree early in life (32).

### Animal Models and Etiological Agents

The characteristic lesions of BA such as the obstruction of the extrahepatic biliary tree and cholestasis, have been successfully reproduced and investigated in several animal models—such as lamb, calf, zebrafish, and mouse. The first three forms of experimental BA in lamb, calf and zebrafish are induced through toxins, while the murine models are achieved upon viral infection (5, 33, 34).

One of the first observations of BA-like pathologies in animals was reported in the Australian outbreak in 1964, 1988, and 2007 when lambs were born with cholestasis after pregnant livestock was exposed to unidentified toxic environmental factors in extreme drought conditions (1, 22, 35), which arose the suspicion that the toxic effect could come from the grass. A group of scientists from the university of Pennsylvania imported a plant species characteristic of that area and used zebrafish bioassays to identify the substance responsible: an isoflavonoid that they named biliatresone (22). This toxic compound, capable of inducing biliary atresia phenotype, is the basis of the theory that implicates hepatotoxins as etiological agents.

The other leading theory about the origin of the disease points toward a viral insult (16, 36). The first implication of an hepatotropic virus as causative factor in BA was suggested by Benjamin Landing (37). Despite the initial contradictory findings regarding the presence and role of reovirus in BA (38–41), numerous viruses have been implicated in the pathology of the disease and evidence of preceding viral infection—MxA proteins (Myxovirus resistance protein 1)—could be found even in the absence of viral material (42–44). Whether the virus is the primary causative factor or an accidental secondary event remains unclear (44, 45).

### Rhesus Rotavirus-Induced Murine Model

**Figure 1:**
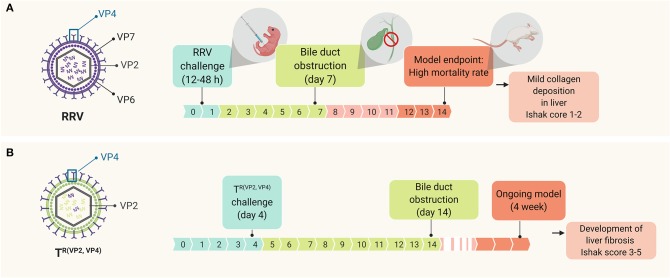
Time line of events in the murine model of BA upon RRV challenging, depicting **(A)** the standard RRV model in comparison with **(B)** the modified model using a novel viral reassortant [T^R(VP2, VP4)^]; this virus reassortant was engineered by replacing the VP2 and VP4 gene of TUCH for the corresponding RRV's VP2 and VP4.

Among all viruses, rhesus rotavirus type A (RRV) is the gold standard to model BA in mice. The use of this murine model has facilitated the study of different aspects of the disease, such as the underlying mechanisms of the pathogenesis (26–28, 46–50) or the identification of novel therapeutic targets (51). This experimental form of BA uses BALB/c newborn mice that, when challenged with RRV within the first hours of life (12–48 h), can recapitulate many aspects of human BA (52) such as time-restricted susceptibility to the viral infection, portal tract infiltration of inflammatory cells and obstruction of both extrahepatic and intrahepatic biliary tree (5, 34). This *in vivo* model allows for the comprehensive study of the early events of the disease that cannot be explored directly in humans, since they happen before the time of diagnosis. However, the RRV model is not yet suitable to study the progression of the disease after duct obstruction, due to the high mortality rate of the mice before the development of liver fibrosis and related long-term complications (5). Previous studies have examined the fibrogenic response in RRV model and observed insufficient fibrosis (Ishak score 1–2) when determined at 2 weeks' time (Figure 1A) (53, 54). These limitations (e.g., high mortality and poor fibrogenic responses), however, could be tackled by optimizing the model induction using reassortant viruses. Recently, a novel RRV-TUCH rotavirus reassortant (TUCH for Tulane University and Cincinnati Children's Hospital) could recapitulate an obstructive jaundice phenotype with lower mortality rates when injected into newborn mice (54). This new model recapitulates the late events of the disease such as liver fibrosis (Ishak score 3–5) and showed a unique resemblance to the human BA, significantly different from CCl_4_ and bile duct ligation models (54) (Figure 1B). This model, therefore, not only improves our current understanding about BA disease pathogenesis but will also contribute toward the identification of new therapeutic targets.

### Other Virus Induced Models

Cytomegalovirus (CMV) has also been used to recapitulate BA in animal models (55). For instance, a regulatory T cell (Treg)-depleted neonatal mouse, when infected with low-dose CMV (LD-CMV) to study BA, induced extensive inflammation, atresia of intrahepatic bile ducts and partial obstruction of the extrahepatic bile ducts. Liver mononuclear cells showed increased percentages of CD3/CD8 T cells and serum autoantibodies (α-enolase) reactive to bile duct epithelial proteins, suggesting the involvement of cellular and humoral autoimmune responses in LD-CMV BA mouse model. There was also an increased hepatic expression of Th1-related genes (tumor necrosis factor α, TNF-α), interferon γ (IFN-γ)-activated genes (signal transducer and activator of transcription 1, STAT-1) and Th1 cytokines/chemokines (lymphotactin, interleukins IL-12p40 and macrophage inflammatory protein 1-alpha, MIP-1α).

### Evidence of Viruses as a Causative Agent of BA

As mentioned earlier, viruses have been proposed as etiological agents in BA. These viruses activate pathways that might predispose certain individuals to develop the disease. In the animal model, the RRV Viral Protein 4 (VP4) gene has been demonstrated to be the major determining factor required for the pathogenesis of BA (49). Rotavirus strains with 87% or more homology to RRV's VP4 were capable of infecting murine bile ducts and inducing the disease as well as activating mononuclear cells, independent of viral titers (56). Further research led to the identification of a key amino acid sequence “SRL” in VP4, a sequence specific to those rotavirus strains that cause obstructive cholangiopathy (57). This tripeptide “SRL” on RRV VP4 was found to bind specifically to the cholangiocyte membrane protein heat shock cognate 70 (Hsc70), defining a novel binding site governing VP4 attachment (57). To gain insight into the mechanisms involved upon VP4-mediated infection, a reverse genetics system was developed to create a mutant of RRV with a single amino acid change in the VP4 protein and compared to that of wild-type RRV (where the arginine “R” in “SRL” region was replaced with glycine “G”) (58). The mutant virus, when injected to mice, demonstrated reduced symptoms and lower mortality in neonatal mice, resulting in an attenuated form of biliary atresia indicating the importance of “SRL” region (57). This “SRL” peptide was also found either on the capsid or the attachment protein of other viruses including reovirus, cytomegalovirus, human papillomavirus, Epstein-Barr virus, bluetongue virus, polyomavirus, coronavirus, respiratory syncytial virus, adenovirus, rodent paramyxovirus, and herpes simplex virus 1. Several of these (cytomegalovirus, Epstein-Barr virus, human papillomavirus, and reovirus) have been detected in explanted livers of infants with BA (59–63). Thus, this sequence in the above-mentioned viruses might be involved in cholangiocyte binding in a similar fashion to the RRV “SRL” peptide. Binding of these viruses to Hsc70 might activate the innate immune system through different pathways. The role of Hsc70 binding in human BA induction as a function of these proteins and their influence in oxidative stress and cell metabolism remain largely unexplored.

## Immunopathogenesis of Biliary Atresia

### Cholangiocyte Immunobiology

Biliary epithelial cells (cholangiocytes) are not only a physical barrier that drains the bile into the duodenum but they are also immunocompetent cells involved in tissue homeostasis, capable of recognizing microbial conserved motifs known as Pathogen Associated Molecular Patterns (PAMPs) through pattern-recognition receptors (PRRs) and initiating an inflammatory response (64–67). Four main families of PRRs have been described, including toll-like receptors (TLRs), retinoic acid inducible gene 1 (RIG-I)-like receptors (RLRs), nucleotide-binding oligomerization domain (NOD)-like receptors (NLRs), and C-type lectin receptors (CLRs) (68).

From the ten types of TLRs that have been identified in mammals, at least 5 of them have been described in mice and human cholangiocytes (64). Among them, TLR-4 is responsible for sensing lipopolysaccharides (LPS) and TLR-3, 7, 8, and 9 are involved in recognition of viral and bacterial RNA or DNA. Activation of these receptors triggers an inflammatory response via Mitogen-activated protein kinases (MAPK), interferon regulatory factor 3 (IRF3) and/or nuclear factor κB (NF-κB) characterized by the production of type I interferons (IFNs) and/or pro-inflammatory cytokines. MAPK signaling is a multifunctional pathway that is pivotal in the innate immune response and viral infection. Among the three central members of the MAPK pathway, extracellular signal-regulated kinase (ERK) 1/2 and p38 activation play the most important roles in RRV infection of cholangiocytes as they seem to be involved in both viral replication and epithelial injury (69). Further studies revealed that ERK phosphorylation and calcium influx appear to be essential to RRV infection, and RRV's viral protein 6 (VP6) drives ERK phosphorylation (70).

**Figure 2:**
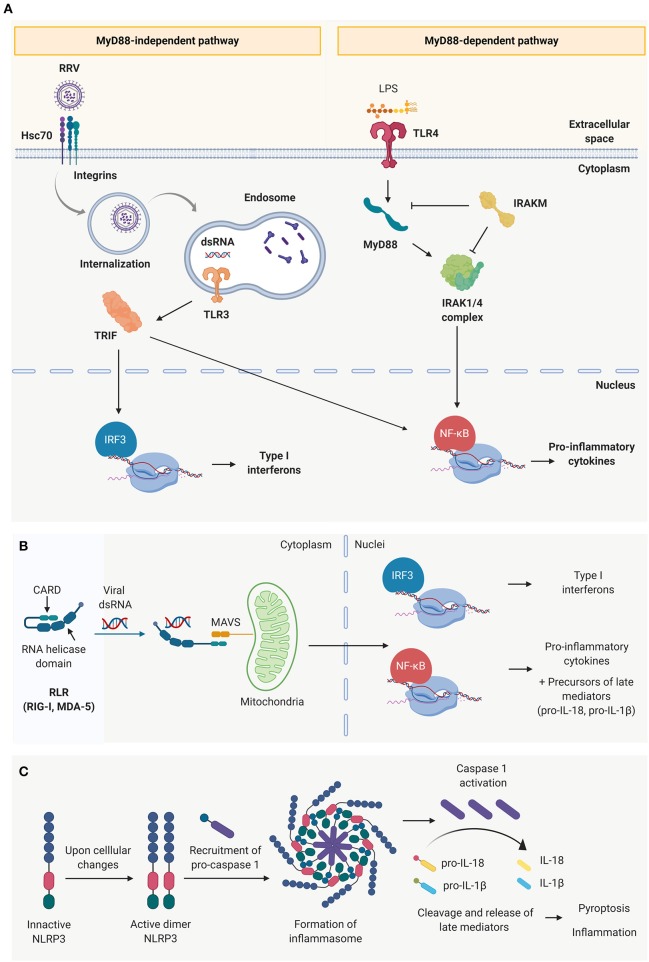
Innate immune receptors present in cholangiocytes. **(A)** Toll-like receptors (TLRs) and schematic representation of the two main signaling pathways: the MyD88 dependent pathway (characteristic of all toll-like receptors except TLR 3) and MYD88 independent pathway (characteristic of TLR3). **(B)** Cytosolic viral sensing of Retinoic-acid-inducible gene I (RIG-I)-like receptors, capable of triggering a pro-inflammatory and antiviral response, and **(C)** nucleotide-binding oligomerization domain (NOD)-like receptors that have the ability to perpetuate the immune response through the formation of inflammasomes, induction of cell death and release of late mediators.

TLRs depend on adaptor molecules– myeloid differentiation primary response 88 (MyD88) or toll/interleukin-1 receptor domain-containing adaptor protein (TRIF)—to effectively initiate and transduce the downstream signal to the nuclei, differentiating them into two main TLR signaling pathways (Figure 2A) (68). In the MyD88-dependent pathway (associated to TLR 1–5, except for TLR-3), the Interleukin-1 receptor-associated kinase (IRAK)-1, −2 and −4 upregulate the production of Type I IFNs and pro-inflammatory cytokines (IL-1β, IL-6, and TNF-α) via MAPK, IRF3, and NF-κB pathways (65, 67, 68). It has been demonstrated that the pathogenesis of murine BA is independent of the MyD88 signaling pathway (71). In MyD88/IRAK-M independent pathway, the activation of TLR-3, 7/8 or 9, associated with the TRIF-dependent signaling, results in the activation of NF-κB and IRF3 signaling cascades (65, 68). This different level of regulation could explain why “endotoxin tolerance” to enteric bacteria can be induced in cultured cholangiocytes by treating them with TLR-4 ligands (like LPS) (72) but “viral tolerance” could not be achieved using the same approach (73).

The RLR family (74) is comprised of cytosolic sensors, including RIG-1 and melanoma differentiation-associated protein 5 (MDA-5) that are capable of binding to dsRNA (75–77). This interaction triggers a conformational change that exposes the two caspase activation and recruitment domains (CARDs) at their N-terminus, which are responsible to recruit the complementary protein mitochondrial antiviral-signaling protein (MAVS) and transduce the signal to the nuclei to produce type I interferons and pro-inflammatory cytokines (Figure 2B) (75, 78). NLRs (e.g., NLRP3), are also cytosolic innate immune receptors that are activated upon recognition of viral dsRNA. Rather than contributing to the initial events of the acute inflammatory response, they amplify the immune response, release late mediators (IL-1β, IL-18 and high mobility group box 1, HMGB-1) and regulate pyroptosis (pro-inflammatory programmed cell death) through the formation of inflammasomes (Figure 2C) (79).

The last group of PRRs described are the large family of CLRs. They are transmembrane receptors, with an immunoreceptor tyrosine-based activation motif (ITAM) or an immunoreceptor tyrosine-based inhibition motif (ITIM), that are able to induce a pro-inflammatory response or modulate it through a crosstalk with other PRRs such as TLRs. CLRs play a crucial role in maintaining immune homeostasis against pathogens and in mounting a pro-inflammatory and/or antiviral response (80–82). Alterations of CLRs have been implicated in different pathological conditions, including gastrointestinal cancers, autoimmune disorders, or allergies (82). It is known that cells from myeloid lineage such as dendritic cells (DCs) and macrophages, as well as some endothelial and epithelial cells, express CLRs; however, it has not been reported in biliary epithelium yet.

Although cholangiocytes play a central role in initiating an immune response upon exposure to the exogenous substances, they are however not capable of mounting an inflammation that is sufficient to induce chemotaxis and recapitulate the obstructing phenotype of BA without the involvement of macrophages and DCs (83–86).

### Mechanisms of Epithelial Injury and Duct Obstruction

**Figure 3:**
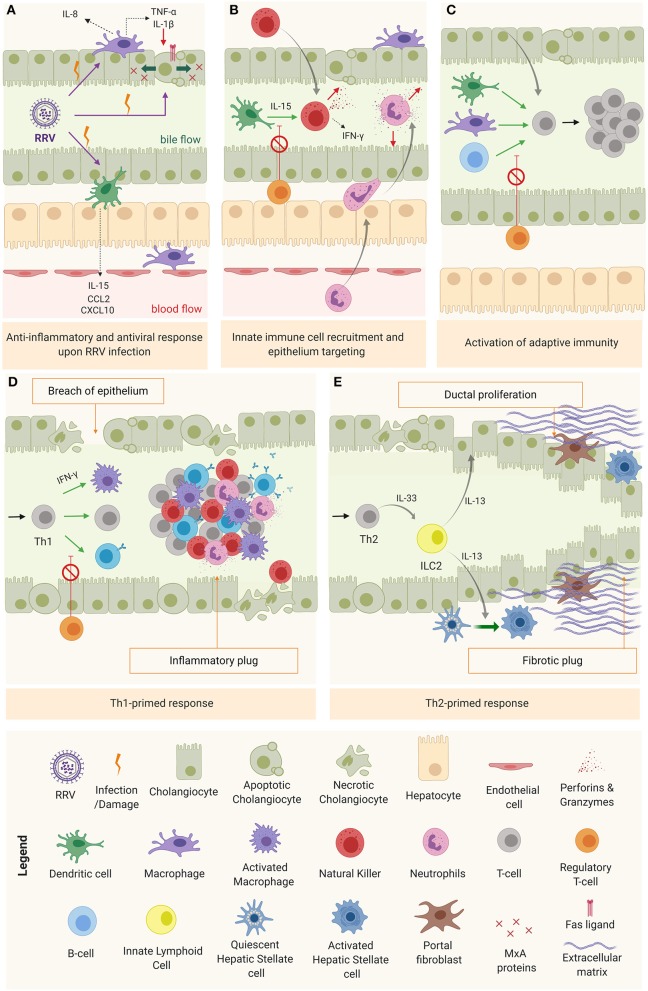
Mechanism of obstruction in biliary atresia. **(A)** RRV infection and activation of the anti-inflammatory and anti-viral response. **(B)** Innate immune cell recruitment & tissue specific attack to epithelia. **(C)** Activation of adaptive immunity **(D)** Th1-primed polarization and alternatively **(E)** Th2 polarization.

Upon viral infection, cholangiocytes, macrophages, and DCs (RRV cellular targets) trigger the anti-viral response through type I interferons in an autocrine and paracrine manner in both infected and surrounding cells to prevent the virus from spreading (5). In infected cells, type I IFNs promote biliary apoptosis by upregulation of tumor necrosis factor related apoptosis ligand (TRAIL) (TNF receptor p55) and CD95 (Fas/Apo1 ligand) (87). In surrounding tissue, IFNs trigger the production of antiviral proteins (Mx) that provide protection against viral infection (Figure 3A) (88). The production of pro-inflammatory cytokines and chemokines by cholangiocytes, macrophages and DCs creates the favorable microenvironment to recruit and activate inflammatory cells, and to promote an immune effector tissue-specific attack (Figure 3B) (84, 85, 89). Among the chemokines produced, the most relevant are IL-8 and IL-15. IL-8, mostly produced by macrophages but also by cholangiocytes (90), recruits and modulates the action of neutrophils (85), basophils, monocytes, and T cells (64, 67, 90); while IL-15, secreted primarily by DCs, attracts and regulates the activity of natural killer (NK), natural killer T (NKT), and gamma-delta cells (89). The recruited inflammatory effector cells are engaged to target specifically the biliary epithelium in a contact dependent manner (91), through IFN-γ-related cytokines (48) and/or cytotoxic agents (perforins, granzymes) (92). Recruited neutrophils produce reactive oxygen species (ROS), leukotrienes, and neutrophil defensins (90). NK cells, activated by DCs via IL-15 (89), induce cholangiocyte death in a contact-dependent manner through Natural killer group 2d (Nkg2d) ligand that interacts with ribonucleic acid export 1 (RAE1) receptors, expressed in infected cells (91) and via the secretion of IFN-γ, perforins, and granzymes (92). In a similar fashion, the cytotoxic power of neonatal CD8^+^ T cells is exerted through cytotoxic agents (perforin, granzymes, IFN-γ) (92) and in a contact-dependent manner by invading the epithelium (27). Mechanistical studies using the RRV-infected BALB/c murine model showed that depletion of NK cells, blockage of the receptor Nkg2d or depletion of CD8^+^ T cells (with impairment of IFN-γ mechanisms) reduced cholangiocyte death, evaded rupture of the epithelium and ultimately prevented the obstruction of the extrahepatic biliary tree (27, 91). Likewise, epithelial integrity was preserved by depleting plasmacytoid DCs or blocking the IL-15 signaling, responsible for NK cell activation (86, 89). These results highlight the specific role of DCs, NK, and CD8^+^T cells in the model.

As the inflammation progresses without being resolved, DCs and macrophages interact chiefly with helper CD4^+^ T cells (Th0) to promote their activation, oligoclonal expansion (93) and differentiation into a specialized phenotype depending on the predominant cytokine microenvironment at the time (Figure 3C). In most of BA patients, this microenvironment is pro-inflammatory (Th1), characterized by IFN-γ production and the activation of effector cells (macrophages, CD8^+^ T cells and B cells) to perpetuate the tissue damage (Figure 3D) (11, 16). In some cases, the infants are not capable of mounting a Th1 response, therefore, the polarization primed is Th2, with IL-13 [produced by type 2 innate lymphoid cells (ILC2)] as a predominant cytokine, responsible for the tissue damage mediated by ductal proliferation and activation of hepatic stellate cell (HSCs) and portal fibroblasts. This is typically the case for the aforementioned cystic variant of BA (94), as depicted in Figure 3E.

### Humoral Immunity

In contrast to T-cell polarization, very little is known about the implication of humoral immunity in the pathogenesis of BA. In the early stage of the disease, humoral-related genes (i.e., immunoglobulins) are transiently suppressed (95). However, B lymphocytes seem to play a role as antigen presenting cells for effector T cell activation as also shown in Figure 3C. An evidence for the role of B lymphocytes has been proposed in a study where the depletion of B-cells in experimental BA was associated with impaired effector T-cell activation and protection against biliary injury (96). Furthermore, humoral duct-specific autoimmunity has been demonstrated in experimental BA (26) but the role of B lymphocytes remains unclear in human BA. Human-based studies regarding humoral activity in BA include the description of immunoglobulins IgM and IgG deposits in the biliary epithelium basement membrane (97) and the detection of autoantibodies (28, 29). Lu et al. (28) detected autoantibodies against α-enolase in the RRV induced mouse model of BA and in serum samples from patients, indicating a role of humoral auto-immunity in disease pathogenesis. The cross-reactivity between an anti-enolase antibody and RRV proteins indicates that molecular mimicry might activate humoral autoimmunity in BA patients. However, further investigation is needed to provide more insight into the implication of humoral immunity in BA.

### Immune Dysregulation

A subset of helper CD4^+^ T cells known as regulatory T cells (Tregs)—that expresses CD25 and forkhead box P3 (FOXP3)—has a pivotal role in immunoregulation and induction of peripheral tolerance. Neonatal Tregs (98, 99) prevent the activation of autoreactive T cells and inhibit the action of several immunocompetent cells (B and T cells, macrophages, dendritic cells, and natural killer cells) (50, 98, 100, 101). In neonatal mice, Tregs populate the spleen from day 3 of life (102) which corresponds the susceptibility time window in the RRV model (100, 103). Moreover, adoptive transfer of Tregs to pups before RRV infection prevented the obstruction of the extrahepatic bile ducts (50, 100, 101). In infants with BA, gene expression of regulatory cytokines (IL-10, transforming growth factor β, TGF-β] and transcription factors (FOXP3) are upregulated in the liver (100), but there is a deficit in number of circulating Tregs in peripheral blood and their regulatory function seems to be impaired (25, 104). Even though the exact underlying mechanisms of Treg malfunctioning and immune dysregulation are not fully understood, epigenetic changes might play a major role. For instance, hypomethylation of FOXP3 promoter was associated with improper functioning of Tregs (25), while hypermethylation of DNA in lymphocytes elicited them to promote an exacerbated inflammatory response (24).

## Mechanisms of Post-Obstruction: Chronic Inflammation, Duct Proliferation, and Fibrosis

**Figure 4:**
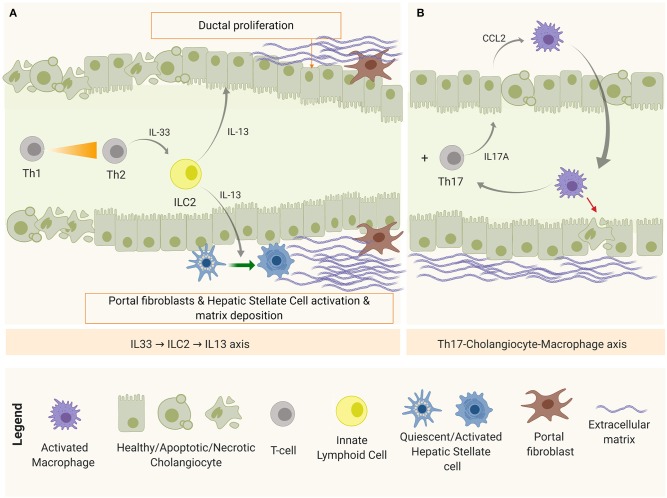
Disease progression mechanisms after bile duct obstruction. **(A)** IL-33-ILC2-IL-13 axis, implicated in fibrosis and duct proliferation and **(B)** Th17-Macrophage axis, as a mechanism of chronic inflammation and damage perpetuation.

After obstruction, regardless of the restoration of the bile flow, the immune-mediated biliary damage persists (9) and the initial Th1-predominant milieu shifts toward a Th2 with the simultaneous emergence of the Th17 subset (Figure 4).

On one hand, apoptotic and necrotic cells release endogenous molecules known as damage-associated molecular patterns (DAMPs)—recognizable by PRRs—as excessive damage or “danger signals” (68). One of these DAMPs is the interleukin IL-33 that, when released by cholangiocytes and hepatocytes, accumulates in the extracellular matrix (ECM) and promotes inflammation and fibrosis. High levels of IL-33 has been detected in serum and tissue biopsies in both patients and experimental BA (105). In this context, IL-33 in the liver is believed to engage with liver-resident innate helper cells (ILC2) that express IL-33 receptor (ST2 or IL-1R4) to produce pro-fibrotic Th2-related cytokines (IL-4, IL-5, IL-9, and IL-13) (106). Among them, IL-13 upregulates the expression of TGF-β and matrix metalloproteinase 9 (MMP9); activates HSCs via IL-4Ra and STAT6, promoting fibrosis in a TGF-β1/SMAD-independent mechanism (107); and stimulates collagen synthesis by myofibroblasts (activated HSCs and portal fibroblasts). Simultaneously, IL-33 was shown to drive duct proliferation in both intra- and extra-hepatic ducts (105). This IL-33-ILC2-IL13 axis is depicted in Figure 4A.

On the other hand, damaged cholangiocytes are shown to produce IL-1β, IL-6, and IL-23 (65). IL-1β, IL-6 are required for Th17 commitment, and IL-23 is needed for the maintenance of this phenotype (108). IL-17A is the representative cytokine of this panel, which induces the production of several pro-inflammatory cytokines and chemokines. Lages et al. identified Th17 cells as the main source of IL-17A after the obstruction of the biliary tree in experimental BA. In this study, a model of biliary injury perpetuation was proposed in which IL-17A stimulated cholangiocytes to produce C-C motif chemokine ligand 2 (CCL2) that recruited inflammatory macrophages expressing IL-17AR to target the epithelium (51), as shown in Figure 4B. In this model, depletion of Th17 cells or blockage of CCL2 prevented bile duct paucity and the number of Th17 cells correlated with the concentration of gamma glutamyl transpeptidase (GGT), a biochemical marker of bile duct injury (51). In BA patients, the presence of Th17 in the biliary tree and peripheral blood has been confirmed, as well as Th17-related markers in liver tissue [IL-17A and retinoic acid-related orphan receptor (ROR)-γt] and serum IL-23. In addition, a high ratio between Th17 and Tregs has been characterized in peripheral blood (109), a trend that has also been observed in chronic liver diseases such as primary biliary cirrhosis (108).

In addition, damaged or pro-apoptotic as well as inflammatory cells (especially Kupffer cells and macrophages) can express or produce hedgehog (Hh) ligands under pathological conditions (110). Cholangiocytes stimulated with Hh ligands (in an autocrine or paracrine manner) produce a wide assortment of cytokines—including IL-6 and TGF-β (111)—and chemokines that attract different populations of inflammatory cells, including neutrophils, monocytes, and lymphocytes (112). Inflammatory cells stimulated by Hh ligands sustain inflammation, while activated HSCs continue to proliferate in response to this stimulus (113). Abnormal over-activation of the Hedgehog pathway has been observed in the context of chronic inflammation-related fibrosis (114, 115), human cholangiopathies (116), and biliary atresia (117). A characteristic Hh ligand in BA is osteopontin (OPN) that has been correlated with severity of the disease (118).

## Macrophages, Microenvironment, and Age-Rage

Like T cells, macrophages can adopt different polarization states depending on the surrounding tissue microenvironment (119). Characterization of these functional programs is important since they seem to have vast implications in the outcome of several chronic auto-inflammatory and degenerative diseases (120). Conventionally, they are divided into classically activated M1 (pro-inflammatory) and alternatively activated M2 (restorative) macrophages (119). Polarization into M1 macrophages is driven by activation of TLR signaling through LPS and IFN-γ challenge; while stimulation with regulatory cytokines (IL-4, IL-10) primes a M2 polarization. Several reports have pointed that, in many contexts, the dichotomy M1/M2 may not be sufficient to describe a relevant macrophage population because of its heterogeneity, the complexity of the activation stimuli, and surrounding tissue microenvironment (121–123). However, in the context of fibrosis, two distinct macrophage population have been described for its role in modulating the body response to chronic injury: pro-fibroinflammatory and resolutive macrophages, often associated with M1 and M2 features, respectively. These polarizations have the ability to influence the tissue microenvironment and with it, the net cellular response and outcome of the disease. For instance, pro-inflammatory macrophages, displaying high levels of inflammatory marker lymphocyte antigen 6 complex, locus C (Ly6C), are characterized by a high production of chemokines (such as CCL2) that attract inflammatory cells to the site of injury, pro-inflammatory cytokines (such as TNF-α and IL-1β) that perpetuate hepatic damage and TGF-β that activates HSCs into ECM-producing myofibroblasts. On the contrary, restorative macrophages, displaying low levels of Ly6C, seem to be responsible for inducing HSCs apoptosis (through TRAIL and MMP9), digesting the excess of ECM and promoting clearance of the profibrotic stimuli, thereby facilitating tissue regeneration (122–125). Both tissue-resident and monocyte-derived macrophages can acquire these functional programs. However, the latter is the predominant population during tissue injury (122), highlighting the relevance of infiltration of inflammatory cells in the course of the disease.

**Figure 5:**
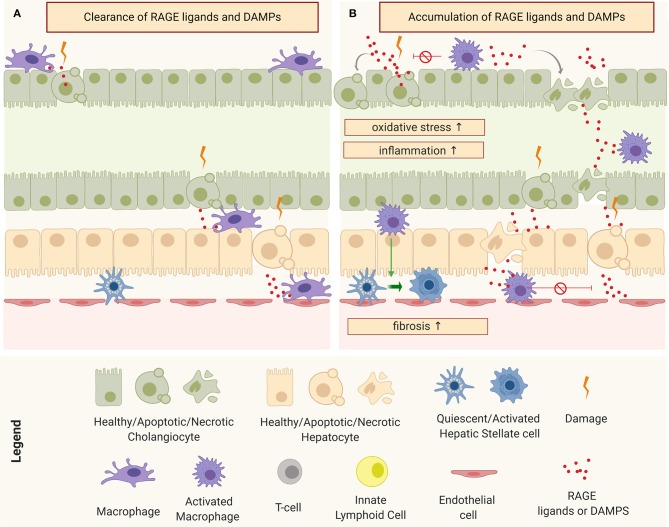
Schematic representation of macrophages and tissue micro-environment: **(A)** Clearance of receptor of advanced glycation end products (RAGE) ligands and Damage-Associated Molecular Patterns (DAMPs) under physiological conditions and **(B)** accumulation of RAGE ligands and DAMPs and consequent perpetuation of damage through the induction of oxidative stress, inflammation, and fibrosis.

Pro-fibroinflammatory macrophages exhibit a wide assortment of mechanisms that allow them to activate and perpetuate inflammation and fibrosis in both TGFβ-dependent and independent circuits. One way to modulate the surrounding cellular response is by influencing the tissue microenvironment. An important component of this microenvironment is the level of oxidative stress, intimately linked to the Advanced Glycation End-Products (AGE)-Receptor of AGEs (RAGE) pathway (120, 122). AGEs refer to a heterogeneous group of toxic by-products that are a result of irreversible non-enzymatic reactions between sugars and proteins as consequence of elevated intra-cellular oxidative species. In normal physiological conditions, AGEs are produced in small amounts, released into the extracellular space, and cleared by specialized phagocytic cells: principally macrophages through scavenger receptors (Figure 5A). However, during chronic injury, under continuous oxidative stress, the production of AGEs is higher than their clearance and this leads to their accumulation in the extracellular space, affecting surrounding cells. Interaction of AGEs (or/and other RAGE ligands, such as S100 proteins and HMGB1) with their receptor triggers a signal transduction cascade through different pathways, resulting in numerous cellular responses such as inflammation, fibrosis, or apoptosis (120, 126, 127), as depicted in Figure 5B.

In the murine model, RRV has the ability to infect the macrophages, resulting in their activation (85). Activated pro-inflammatory macrophages are one of the main sources of AGEs but damaged cholangiocytes and hepatocytes have also been shown to produce several RAGE ligands in response to injury. In patients with BA, the serum levels of soluble RAGE has been correlated with the severity of the disease (128). A recent network analysis study involving the three main human cholangiopathies (including BA), identified a common connectome in which AGE-RAGE pathways occupy central nodes (129). Remarkably, we have observed an induction of oxidative species and production of AGE-RAGE ligands in RRV-infected cholangiocytes (unpublished work), which suggests an involvement of oxidative stress circuits from the onset of the disease.

## Therapeutics and Clinical Trials

The routine treatments of BA patients after HPE are ursodeoxycholic acid, antibiotics, and fat-soluble vitamin formulations that have not substantially improved the outcomes of the disease. In a double-blind, placebo-controlled study (START trial) corticosteroid administration within 3 days of the HPE did not change the outcome of the BA cohort while increased the risk of serious adverse effects as compared to placebo controls (130). Although corticosteroids in BA infants younger than 2 weeks of age did appear to improve biliary drainage, with pending data on native liver survival (131) suggesting a possibility of corticosteroids use on these subsets of infants. In the future, the agents which are currently being tested in cholestatic and fibrotic liver diseases in adults (132) can also be investigated in BA, such as the farnesoid X receptor (FXR) agonist, obeticholic acid, and the modified bile acid norursodeoxycholic acid, which are also currently used in primary biliary cholangitis (PBC) and primary sclerosing cholangitis (PSC) patients (133, 134). Other agent such as apical sodium-dependent bile acid transporter (ASBT) inhibitor may reduce bile acid burden in the liver. The two other agents that are currently used in clinics for pediatric liver diseases—bile acid sequestrants (cholestyramine or colesevelam) and ursodeoxycholic acid—are yet to be thoroughly tested in clinical trials in BA (135).

## Conclusion and Future Prospective

Due to the establishment of experimental models of BA, especially the RRV murine model, some of the driving mechanisms of epithelial injury and duct obstruction have been elucidated, and the corresponding key cellular and molecular targets have been identified. However, the real applicability of these targets for therapy is hindered due to the lack of early diagnosis and screening tools, and that many questions regarding the etiology of the disease remain unanswered. The molecular and cellular mechanisms in which the disease progresses are still under investigation. Increasing evidence suggests a deeper implication of intricated mechanisms of the innate immunity from the onset of the disease: namely, oxidative stress, altered metabolism, and induction of long-term/abnormal epigenetic changes. Among them, AGE-RAGE pathway has attracted most of the attention since it encompasses key circuits involved in the pathogenesis of several chronic inflammatory and degenerative diseases, including biliary atresia. Further investigation is needed to determine the extent of implication of the AGE-RAGE pathway and its crosstalk with other fibro-inflammatory circuits. Because macrophages are one of the main drivers of AGE-RAGE and their functional polarizations seem to occupy a central role in the modulation of the tissue response and outcome in chronic conditions, future research should interrogate these cell populations in the context of biliary atresia. Imperatively, there is a need to develop new or improve existing experimental platforms to perform mechanistical studies of later events of the disease and facilitate the identification and implication of cell populations and pathways. In addition, deeper understanding of the model induction through other viruses and/or toxins could shed some light into the etiology of the disease and aid the development of new therapies to manage BA patients without the need of surgery.

## Author Contributions

AO-P drafted the manuscript. BD, HT, RB, GT, and SM supported the writing of the manuscript, implemented it, and ensured scientific quality. AO-P, RB, and SM designed the figures. AO-P, RB, GT, and SM made the final corrections. All authors corrected and approved the manuscript.

### Conflict of Interest

The authors declare that the research was conducted in the absence of any commercial or financial relationships that could be construed as a potential conflict of interest.
